# Diabetes Affects the Pituitary Adenylate Cyclase-Activating Polypeptide (PACAP)-Like Immunoreactive Enteric Neurons in the Porcine Digestive Tract

**DOI:** 10.3390/ijms22115727

**Published:** 2021-05-27

**Authors:** Katarzyna Palus, Michał Bulc, Jarosław Całka, Łukasz Zielonka, Marcin Nowicki

**Affiliations:** 1 Department of Clinical Physiology, Faculty of Veterinary Medicine, University of Warmia and Mazury in Olsztyn, Oczapowskiego Str. 13, 10-718 Olsztyn, Poland; michal.bulc@uwm.edu.pl (M.B.); calkaj@uwm.edu.pl (J.C.); 2 Department of Veterinary Prevention and Feed Hygiene, Faculty of Veterinary Medicine, University of Warmia and Mazury in Olsztyn, Oczapowskiego Str. 13, 10-719 Olsztyn, Poland; lukasz.zielonka@uwm.edu.pl; 3 Institute of Anatomy, University of Leipzig, Liebigstraße 13, D-04103 Leipzig, Germany; Marcin.Nowicki@medizin.uni-leipzig.de

**Keywords:** diabetes, enteric nervous system, gastrointestinal tract, PACAP, pig

## Abstract

Diabetic gastroenteropathy is a common complication, which develops in patients with long-term diabetes. The pituitary adenylate cyclase-activating polypeptide (PACAP) is a neuropeptide known for its cytoprotective properties and plays an important role in neuronal development, neuromodulation and neuroprotection. The present study was designed to elucidate, for the first time, the impact of prolonged hyperglycaemia conditions on a population of PACAP-like immunoreactive neurons in selected parts of the porcine gastrointestinal tract. The experiment was conducted on 10 juvenile female pigs assigned to two experimental groups: The DM group (pigs with streptozocin-induced diabetes) and the C group (control pigs). Diabetes conditions were induced by a single intravenous injection of streptozocin. Six weeks after the induction of diabetes, all animals were euthanised and further collected, and fixed fragments of the stomach, duodenum, jejunum, ileum and descending colon were processed using the routine double-labelling immunofluorescence technique. Streptozotocin-induced hyperglycaemia caused a significant increase in the population of PACAP-containing enteric neurons in the porcine stomach, small intestines and descending colon. The recorded changes may result from the direct toxic effect of hyperglycaemia on the ENS neurons, oxidative stress or inflammatory conditions accompanying hyperglycaemia and suggest that PACAP is involved in regulatory processes of the GIT function in the course of diabetes.

## 1. Introduction

Diabetes mellitus (DM) is a group of metabolic diseases characterised by hyperglycaemia resulting from defects in insulin secretion, insulin action (or both) and is one of the most commonly encountered endocrinopathies worldwide. The International Diabetes Federation (IDF) reports that there were 424 million cases of diabetes in 2017. It is estimated that the number of cases in 2040 will increase significantly and will amount to 642 million [1]. Previous studies have shown that hyperglycaemia evokes chronic complications by vascular endothelium damage, inflammatory conditions and several organic dysfunctions [2]. Diabetic gastroenteropathy is a common complication that develops in patients with long-term diabetes. Clinical symptoms including abdominal pain, vomiting, heartburn, diarrhoea and obstipation have been confirmed in diabetic patients, especially in poorly controlled glycaemia [2,3]. Although gastroenteropathy contributes to lowering the quality of life and hinders proper glycaemic control, knowledge of its pathophysiology is still very fragmented. There is evidence suggesting that autonomic neuropathy is the main reason for these disturbances [4].

The enteric nervous system (ENS) is one of the parts of the autonomic nervous system located in the wall of the gastrointestinal tract, which is characterised by a multitude of neurons in its composition and an extraordinary richness of neurotransmitters [5]. These features allow ENS considerable autonomy in the control of many important functions of the digestive tract, such as motor activity, secretion and circulation processes as well as local inflammatory conditions without the participation of the central nervous system (CNS) [5,6]. The structure and arrangement of individual plexuses in particular parts of the gastrointestinal tract (GIT) depend on the species. In large mammals, two types of plexuses can be found in the oesophagus and stomach: Myenteric plexus (MP)—responsible mainly for the regulation of motor activity and submucous plexus (SP)—controlling secretory processes. In the further parts of the GIT (small and large intestines), there are two submucous plexuses: Inner submucous plexus (ISP) and outer submucous plexus (OSP) [6,7]. It should also be emphasised that changes in the neurochemical characteristics of ENS neurons in response to pathological stimuli are an important element of the so-called neuronal plasticity [6]. In light of previous research, neuroactive substances synthesised and released by ENS neurons may be an important element of the protective mechanism against harmful factors like hyperglycaemia [6,7,8].

The pituitary adenylate cyclase-activating polypeptide (PACAP) is a neuropeptide, isolated for the first time from an ovine hypothalamus extract [9]. Two biologically active forms, a 38-amino-acid-peptide (PACAP-38) and the N-terminal 27-amino-acid peptide (PACAP-27), are known so far [9,10]. PACAP shows high sequence homology with vasoactive intestinal peptide (VIP) and is classified as a member of the VIP/glucagon/secretin superfamily [10]. PACAP is widely expressed in the peripheral and central nervous system (CNS) [10,11]. The presence of PACAP was also confirmed in the digestive system, including neuroendocrine cells, intramural neurons and nerve fibres, the pancreas and the liver, where it regulates the secretion of digestive juices, smooth muscle contraction, cell migration and proliferation [6,12,13,14,15]. In addition, PACAP is known for its cytoprotective properties and plays an important role in neuronal development, neuromodulation and neuroprotection [16]. Accumulating evidence indicates the anti-apoptotic, anti-inflammatory and antioxidant effects of PACAP in many experimental models of inflammatory and degenerative diseases [6,13,16,17]. Furthermore, recent studies provide evidence that PACAP has a positive effect on glucose-stimulated insulin secretion and glucose tolerance, stimulates proliferation of beta-cells and may prevent diabetes-related organ complications, such as micro- and macroangiopathy, retinal dysfunction, neuropathy and insufficient insulin secretion [18,19].

Animal models play a crucial role in the detection and characterisation of disease pathophysiology and target identification in the study of new therapeutic agents in vivo. The pig is an omnivorous species whose gastrointestinal anatomy and physiological processes are very similar to those of humans and is a widely used research model for studying disorders of the GIT [20]. Streptozotocin (STZ) is one of the most potent diabetogenic chemicals used in the induction of diabetes. STZ, as a powerful alkylating agent, leads to deoxyribonucleic acid breaks in the beta cells, resulting in the induction of insulinopoenic diabetes [21]. Pigs with streptozotocin-induced diabetes appear to be a very useful animal model in studies designed to assess the impact of hyperglycaemia on ENS neurons. Thus, the present study was designed to elucidate, for the first time, the impact of prolonged hyperglycaemia conditions on a population of PACAP-like immunoreactive (LI) neurons in selected parts of the porcine gastrointestinal tract.

## 2. Results

### 2.1. Control of Glycaemia

**Figure 1:**
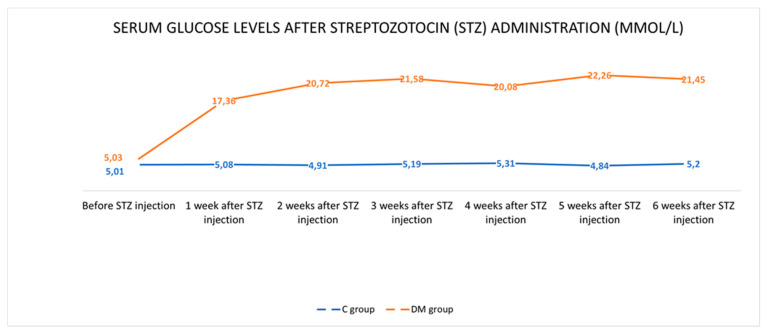
Serum glucose levels in control and diabetic pigs during the experiment.

Before the administration of STZ, the mean value of serum glucose level was comparable in both animal groups and was within the reference standards for this species (5.01 ± 0.10 mmol/L in the C group and 5.03 ± 0.10 mmol/L in the DM group, respectively). Throughout the experiment, the blood glucose level in the control pigs remained constant and averaged 5.09 ± 0.14 mmol/L. In turn, in pigs from the DM group after STZ injection, an increased level of glucose in the blood was observed (Figure 1). Detailed data were described previously by Bulc at al. [8]. In general, hyperglycaemia persisted for six weeks until euthanasia of the animals and the average glucose level in the blood was 20.58 ± 0.55 mmol/L. Despite the hyperglycaemia, the animals were in good general condition and the use of insulin was not required.

### 2.2. Immunofluorescence Technique

#### 2.2.1. PACAP-LI Neurons in the Porcine Corpus of the Stomach

**Figure 2:**
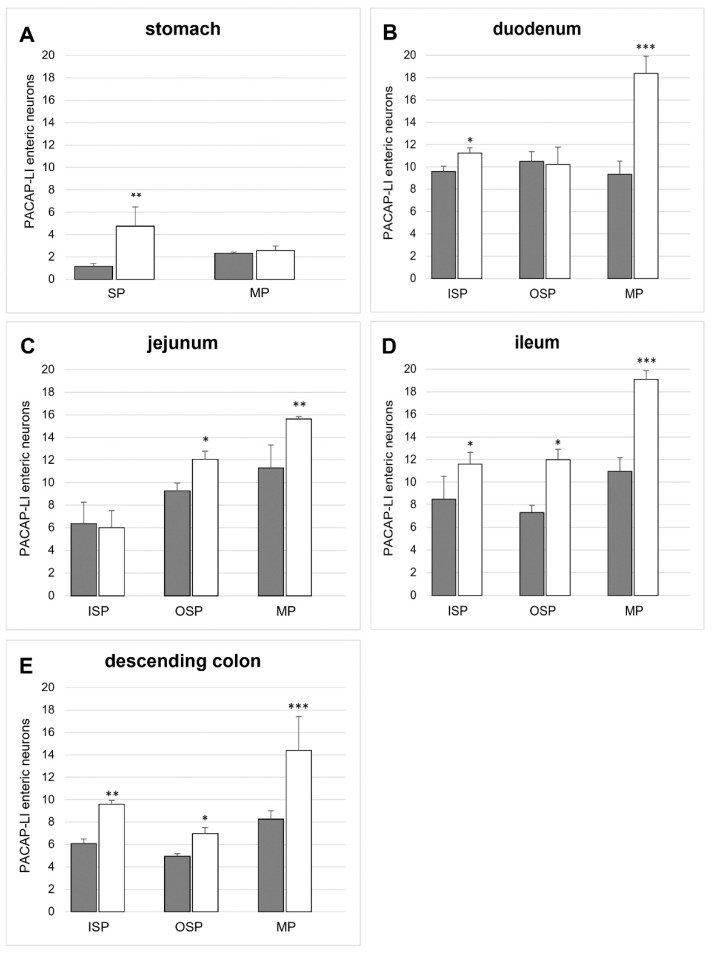
Histograms showing the percentage of PACAP- LI neurons in the wall of selected parts of the gastrointestinal tract in control (grey bars) and diabetic pigs (white bars). (**A**)—PACAP-LI enteric neurons in the corpus of the stomach, (**B**)—PACAP-LI enteric neurons in the duodenum, (**C**)—PACAP-LI enteric neurons in the jejunum, (**D**)—PACAP-LI enteric neurons in the ileum, (**E**)—PACAP-LI enteric neurons in the descending colon. Significant differences were assessed with Student’s t-test for independent samples (* *p* < 0.05, ** *p* < 0.01, and *** *p* < 0.001).

**Figure 3:**
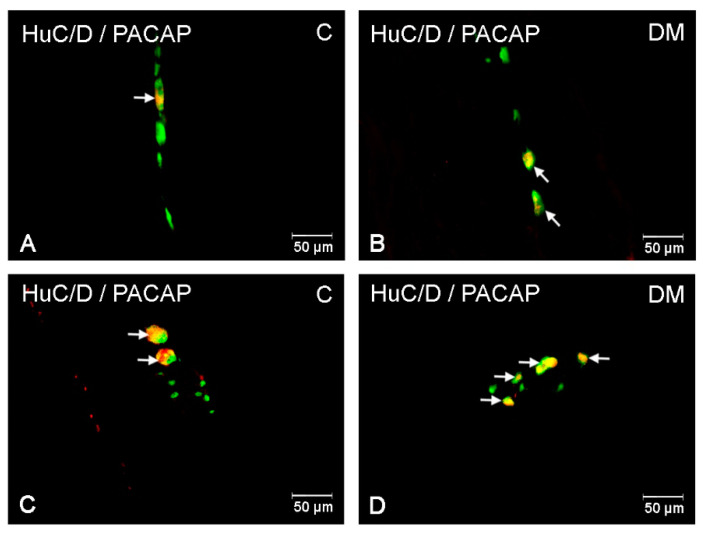
PACAP-LI enteric neurons in the porcine corpus of the stomach. (**A**)—neurons immunoreactive to HuC/D (panneuronal marker) and PACAP in the myenteric plexus of control pigs; (**B**)—neurons immunoreactive to HuC/D (panneuronal marker) and PACAP in the myenteric plexus of diabetic pigs, (**C**)—neurons immunoreactive to HuC/D (panneuronal marker) and PACAP in the submucous plexus of control pigs; (**D**)—neurons immunoreactive to HuC/D (panneuronal marker) and PACAP in the submucous plexus of diabetic pigs. All pictures were created by digital superimposition of two colour channels (green for HuC/D and red for PACAP).

In the control pigs, the population of PACAP-LI intramural neurons located in the corpus was sparse. In the myenteric plexus (MP), PACAP-LI neurons constituted 2.33 ± 0.12% of HuC/D-positive neurons (Figure 2A and Figure 3A). Similarly, in the submucous plexus (SP), only 1.15 ± 0.26% of neurons showed immunoreactivity to PACAP (Figure 2A and Figure 3C). Hyperglycaemia led to an increase in the number of PACAP-LI neurons in the SP (to 4.74 ± 1.72 %) in the corpus of the stomach (Figure 2A and Figure 3D). However, in the MP, the value was similar to that observed in the control group (Figure 2A and Figure 3B).

#### 2.2.2. PACAP-LI Neurons in the Porcine Small Intestine

**Figure 4:**
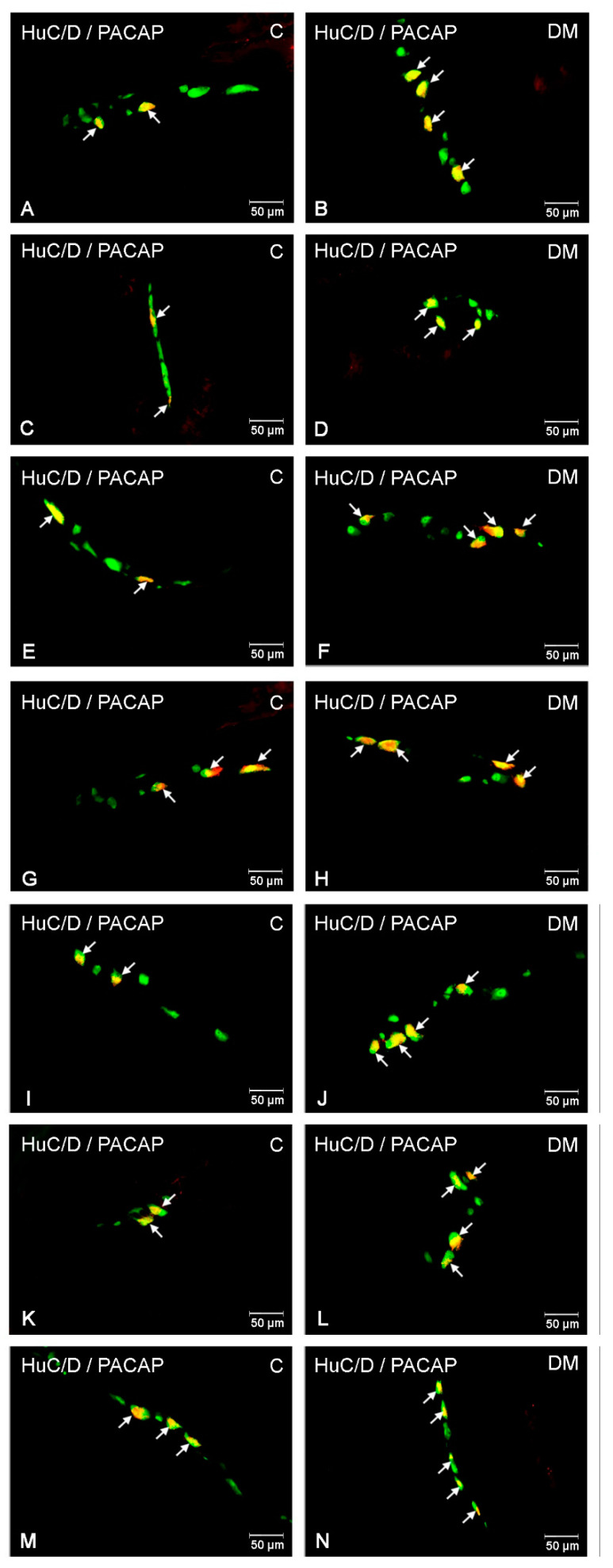
PACAP-LI enteric neurons in the porcine small intestine. (**A**)—neurons immunoreactive to HuC/D (panneuronal marker) and PACAP in the myenteric plexus in the duodenum of control pigs; (**B**)—neurons immunoreactive to HuC/D (panneuronal marker) and PACAP in the myenteric plexus in the duodenum of diabetic pigs, (**C**)—neurons immunoreactive to HuC/D (panneuronal marker) and PACAP in the inner submucous plexus in the duodenum of control pigs; (**D**)—neurons immunoreactive to HuC/D (panneuronal marker) and PACAP in the inner submucous plexus of diabetic pigs; (**E**)—neurons immunoreactive to HuC/D (panneuronal marker) and PACAP in the myenteric plexus in the jejunum of control pigs; (**F**)—neurons immunoreactive to HuC/D (panneuronal marker) and PACAP in the myenteric plexus in the jejunum of diabetic pigs; (**G**)—neurons immunoreactive to HuC/D (panneuronal marker) and PACAP in the outer submucous plexus in the jejunum of control pigs; (**H**)—neurons immunoreactive to HuC/D (panneuronal marker) and PACAP in the outer submucous plexus in the jejunum of diabetic pigs; (**I**)—neurons immunoreactive to HuC/D (panneuronal marker) and PACAP in the myenteric plexus in the ileum of control pigs; (**J**)—neurons immunoreactive to HuC/D (panneuronal marker) and PACAP in the myenteric plexus in the ileum of diabetic pigs; (**K**)—neurons immunoreactive to HuC/D (panneuronal marker) and PACAP in the outer submucous plexus in the ileum of control pigs; (**L**)—neurons immunoreactive to HuC/D (panneuronal marker) and PACAP in the outer submucous plexus in the ileum of diabetic pigs; (**M**)—neurons immunoreactive to HuC/D (panneuronal marker) and PACAP in the inner submucous plexus in the ileum of control pigs; (**N**)—neurons immunoreactive to HuC/D (panneuronal marker) and PACAP in the inner submucous plexus in the ileum of diabetic pigs. All pictures were created by digital superimposition of two colour channels (green for HuC/D and red for PACAP).

In the small intestine, PACAP-positive neurons were observed in all of the studied enteric plexuses and parts of the intestine. In the duodenum, the most numerous population of PACAP-LI neurons was noted in the OSP (10.50 ± 0.88%) (Figure 2B), with slightly smaller populations in the ISP (9.60 ± 0.46%) (Figure 2B and Figure 4C) and the MP (9.35 ± 1.18%) (Figure 2B and Figure 4A). In the jejunum, a higher number of PACAP-positive cell bodies were detected in the MP (11.29 ± 2.02%) (Figure 2C and Figure 4E) and slightly fewer were detected in the OSP (9.25 ± 0.70%) (Figure 2C and Figure 4G) and the ISP (6.36 ± 1.89%) (Figure 2C). In turn, in the ileum, PACAP-LI neurons accounted for 10.96 ± 0.98 % neurons in the MP (Figure 2D and Figure 4I), 8.50 ± 0.64 % in the ISP (Figure 2D and Figure 4M) and 7.32 ± 1.21 % in the OSP (Figure 2D and Figure 4K), respectively.

Streptozotocin-induced diabetes evoked alterations in the number of PACAP-LI intramural neurons in the porcine small intestine (Figure 2). The severity of the changes depended on the type of plexus examined and its location. In the duodenum, a large increase in the population of PACAP-LI neurons was observed in the MP (to 18.39 ± 2.10 %) (Figure 2B and Figure 4B) and a slightly smaller population was observed in the ISP (to 11.25 ± 0.45%) (Figure 2B and Figure 4D). In the jejunum, an increase was detected in the MP (to 15.64 ± 0.20%) (Figure 2C and Figure 4F) as well as in the OSP (to 12.05 ± 0.74) (Figure 2C and Figure 4H). In the ileum, the increase was statistically significant in all types of intramural plexuses (to 19.10 ± 0.98%) in the MP (Figure 2D and Figure 4J), to 11.98 ± 0.93% in the OSP (Figure 2D and Figure 4L) and to 11.60 ± 1.02 % in the ISP (Figure 2D and Figure 4N), respectively.

#### 2.2.3. PACAP-LI Neuron in the Porcine Descending Colon

**Figure 5:**
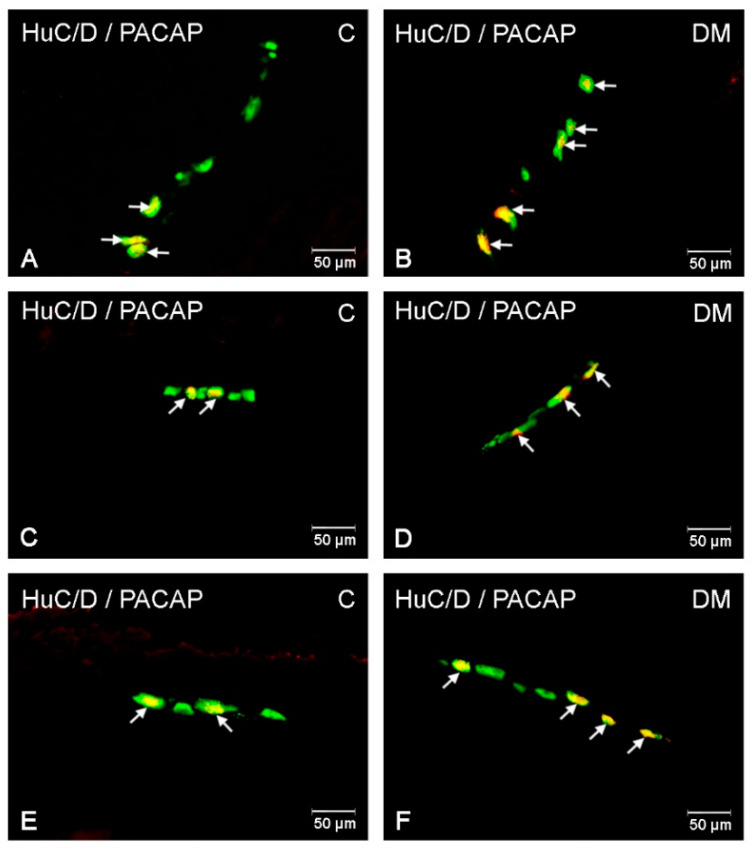
PACAP- LI enteric neuron in the porcine descending colon. (**A**)—neurons immunoreactive to HuC/D (panneuronal marker) and PACAP in the myenteric plexus in the descending colon of control pigs; (**B**)—neurons immunoreactive to HuC/D (panneuronal marker) and PACAP in the myenteric plexus in the descending colon of diabetic pigs; (**C**)—neurons immunoreactive to HuC/D (panneuronal marker) and PACAP in the outer submucous plexus in the descending colon of control pigs; (**D**)—neurons immunoreactive to HuC/D (panneuronal marker) and PACAP in the outer submucous plexus in the descending colon of diabetic pigs; (**E**)—neurons immunoreactive to HuC/D (panneuronal marker) and PACAP in the inner submucous plexus in the descending colon of control pigs; (**F**)—neurons immunoreactive to HuC/D (pan-neuronal marker) and PACAP in the inner submucous plexus in the descending colon of diabetic pigs. All pictures were created by digital superimposition of two colour channels (green for HuC/D and red for PACAP).

In the descending colon, PACAP-LI neurons constituted 8.26 ± 0.76% of HuC/D-positive neurons in the MP (Figure 2E and Figure 5A), 4.96 ± 0.22% in the OSP (Figure 2E and Figure 5C) and 6.07 ± 0.42% in the ISP (Figure 2E and Figure 5E), respectively. Long-term hyperglycaemia triggered an increase in the number of PACAP-positive neurons in all types of intramural neurons under investigation (Figure 2). The most significant increase was noted in the MP (to 14.40 ± 3.04%) (Figure 2E and Figure 5B) and a slightly lower increase was recorded for the OSP (to 6.98 ± 0.53%) (Figure 2E and Figure 5D) and the ISP (to 9.59 ± 0.35%) (Figure 2E and Figure 5F).

## 3. Discussion

In the present study, for the first time, the influence of streptozotocin-induced diabetes on PACAP- like immunoreactive enteric neurons was demonstrated. Enteric neurons showing immunoreactivity to PACAP were visualised in each submucous (SP, ISP and OSP) and myenteric plexuses in the wall of the entire studied GIT (the corpus of the stomach, small intestines and descending colon). The obtained results are in agreement with previous findings in mammals showing PACAP immunoreactivity in enteric neurons and nerve fibres along the entire length of the digestive tract [6,12,13,14,15]. The regional distribution of PACAP in particular sections of the GIT and innervation density varies somewhat between species [6,12,13,14,15]. Nevertheless, the presence of PACAP in enteric neural structures supports previous reports on the significant role of this neuropeptide in the physiology of the alimentary tract. PACAP, together with VIP, as a non-cholinergic, non-adrenergic inhibitory neurotransmitter, induces relaxation of smooth muscle in the gastrointestinal wall [22]. Additionally, several studies have shown that PACAP stimulates pancreatic secretion, hormone release, gastric secretion and active ion transport in the intestine [13,23]. PACAP demonstrates its biological effect by binding to three G-protein-coupled receptor subtypes: VPAC1, VPAC2 and PAC1. The PAC1 receptor is considered to be a PACAP-specific receptor and binds PACAP with high affinity [11]. The presence of PAC1 receptors in the digestive tract has been described in various animals and humans [11,24,25,26]. Additionally, PAC1 immunoreactivity has been shown in the rat gastric and colonic myenteric neurons [25], which confirmed PACAP engagement in neuronal regulation of the GIT function.

Diabetic gastroenteropathy, as a complication of long-term and inadequately controlled glycaemia in the course of diabetes mellitus, is an increasingly recorded problem that leads to a significant deterioration in patient life quality [2]. The pathophysiology of this disorder varies and depends on organs and symptoms. It has been shown that hyperglycaemia leads to autonomic neuropathy, mainly concerning the vagus nerve but also other parts of the peripheral nervous system [4]. Other authors have reported a loss of Interstitial Cell of Cajal (ICC), forming a gastric muscle pacemaker which is manifested by disorders of gut motility, including dysphagia, gastroparesis and obstructions [2,27]. Motility disorders are also a result of myenteric plexus neuropathy, physiologically involved in the control of smooth muscle activity in the alimentary tract [28]. Although no clinical symptoms of gastroenteropathy were observed in diabetic pigs, we may suspect that the high glucose levels may evoked dysfunction of ENS neurons. An increased number of PACAP-LI enteric neurons in each part of the GIT observed in the present study may be a response to the neurotoxic effect of hyperglycaemia on the ENS neurons. PACAP is known for its neuromodulatory and neuroprotective properties [16]. It is involved in neuronal proliferation and differentiation and axonal growth and development of glial cells [16,17,18]. Increased expression of PACAP in the CNS has been noted in disorders caused by neurotoxic agents, such as ethanol [29], kainic acid [30], oxidative-related factors [31], beta-amyloid peptide [32] and glucotoxicity [33]. In the GIT, the cytoprotective effects of PACAP in experimentally induced small bowel ischemia and transplantation have been reported [34]. Additionally, increased immunoreactivity of PACAP in ENS structures has been shown during nerve injury [6], NLPZ administration [35] and zearalenone intoxication [36].

It is also worth mentioning that an imbalance between pro- and anti-oxidative factors leading to oxidative stress in the course of diabetes is often reported. As a result, neuronal damage occurs, including a decrease in the density and diameter of axons, degeneration of Shwann’s cells and changes in endoneurial vascularisation and cell apoptosis [2]. Additionally, in long-term diabetes, insulin-growth factor I (IGF-I) is reduced, which leads to atrophic changes in smooth muscles, resulting in an impaired GIT function [37]. The antioxidative effect of PACAP has been identified in various culture studies in vitro and in various animal models in vivo [10,16,31,38]. Studies of PACAP-deficient mice have shown that endogenous PACAP plays a crucial role in reducing oxidative stress and its deficiency leads to severe oxidative damage [38]. Kasica et al. [39] showed that PACAP has an anti-apoptotic effect on zebrafish hair cells by reducing the cleaved caspase-3 level and reducing oxidative stress. Furthermore, the antioxidative effect of PACAP on small intestine INT 407 cells was also shown [40].

It is also likely that the increase in PACAP immunoreactivity in ENS neurons may be due to local inflammatory conditions that often accompany diabetes [1,2,3]. Accumulating evidence indicates that PACAP plays an important role in immunity and inflammation [10,11,12,16,34]. In experimental ileitis, PACAP exerts an immunomodulatory role through the decreased activity of T lymphocytes, increased synthesis of anti-inflammatory cytokines and reduction of oxidative stress [41]. Similarly, in dextran sodium sulphate-induced colitis, PACAP regulates the levels of inflammatory cytokines [42]. Later, anti-inflammatory and cytoprotective effects of the neuropeptide were confirmed in an intestinal autotransplantation model [34]. Additionally, Gonkowski and Całka [6] showed that natural and chemically induced inflammation leads to an increase in PACAP immunoreactivity in the wall of the descending colon. Moreover, gastrointestinal disturbances in diabetic patients are often accompanied by visceral pain [2]. The augmentation of PACAP-27 synthesis observed in the present study correlates well with the fact that PACAP is a sensory neurotransmitter. Earlier studies demonstrated that PACAP participates in the transmission of nociceptive stimuli during different pathological states [6,16].

It is also worth noting that although we generally observed an increase in the population of PACAP-LI neurons in pigs with STZ-induced diabetes, these changes differed in the individual plexus and parts of the GIT studied. It has been shown that the role of PACAP in the GIT is multifunctional and organ-dependent. Their biological role in particular fragments of the GIT is also dependent on the interaction with the specific type of receptor [6,43]. PACAP as an inhibitory neurotransmitter elicits a dose-dependent relaxation of smooth muscle, especially in the oesophagus, stomach and large intestine [14,15,43]. It is also a strong regulatory factor engaged in the control of gastric acid secretion, hormone and neurotransmitter release and the proliferation of enterochromaffin-like cells [6,15,43]. An increased number of PACAP-LI neurons in MP observed in the present study suggests that PACAP may be involved in the control of motor function of the small intestines and the descending colon in the course of diabetes. Furthermore, the increased expression of PACAP in the submucous plexuses may result from its participation in the control of the secretory functions of the particular parts of the GIT under hyperglycaemia.

## 4. Materials and Methods

The experiment was conducted on 10 juvenile female pigs (the White Large Polish breed, about 20 kg of body weight [b.w.]). After one week of acclimatisation prior to the experimental procedures, pigs were randomly and equally (five pigs per group) assigned to two experimental groups, including the DM group (pigs with streptozocin-induced diabetes) and the C group (control pigs). Diabetes conditions were induced by a single intravenous injection of streptozocin (STZ) (150 mg/kg of b.w., Sigma-Aldrich, St. Louis, MO, USA, S0130) as described previously by Bulc et al. [8]. To avoid episodes of sudden hyperglycaemia induced by STZ administration, animals from the DM group received 250 mL of 50% glucose solution per animal. In turn, pigs from the control group received only citrate buffer (a solvent for STZ). All procedures on animals were conducted according to the Act for the Protection of Animals for Scientific or Educational Purposes of 15 January 2015 (Official Gazette 2015, No. 266), applicable in the Republic of Poland, and were approved by the Local Ethical Committee in Olsztyn (decision number 13/2015/DTN, 30. 10. 2015). During the experiment, pigs had constant access to water and were fed twice a day (morning and evening). Blood for glucose tests was collected from a capillary on the ear by an experienced veterinarian before morning feeding. For this purpose, pigs were restrained in accordance with the procedure prescribed for this species. Immediately after blood sampling, the blood glucose level was determined by colorimetric measurement of the glucose concentration using an Accent-200 biochemical analyser (Germany) (wavelength: 510 nm/670 nm). Measurements were made in both groups before the start of the experimental procedures, 48 h after the administration of STZ and then once a week for the duration of the experiment.

On day 43 of the experiment (six weeks after the induction of diabetes with STZ), all animals were euthanised by intravenous administration of pentobarbital (Vetbutal, Biowet, Puławy, Poland) and then transcardially perfused with 4% buffered paraformaldehyde (pH 7.4). Immediately after perfusion, the following tissues were collected for further research: The corpus of the stomach, small intestines (duodenum, jejunum and ileum) and the descending colon. The samples were then post-fixed in the same fixative (10 min), rinsed in phosphate buffer (pH 7.4) for 2 days with daily buffer change and finally placed in an 18% buffered sucrose solution.

In the next step, 12-μm-thick cryostat sections of the tissue samples were processed using the routine double-labelling immunofluorescence technique, as described previously by Palus et al. [44]. In brief, after air-drying at room temperature for 45 min, the sections were rinsed three times (10 min) in 0.1 M phosphate-buffered saline (PBS, pH 7.4), blocked with a 10% normal goat serum in PBS with 0.3% Triton X-100 (Sigma, St. Louis, MO, USA) and 1% bovine serum albumin (BSA; Sigma, St. Louis, MO, USA) for one hour, rinsed three times in PBS (10 min) and finally incubated overnight at room temperature with primary antisera raised against Hu C/D (mouse polyclonal, Invitrogen, Waltham, MA, USA, Cat. No. A-21271, working dilution: 1:1000 used as a pan-neuronal marker) and pituitary adenylate cyclase-activating peptide (PACAP, guinea pig polyclonal, Peninsula, San Carlos, CA, USA, Cat. No. T-5039, working dilution: 1:3000). On the next day, the sections were rinsed three times in PBS (10 min) and incubated with a mixture of the secondary antibody (Alexa Fluor 488 nm donkey anti-mouse, ThermoFisher Scientific, Waltham, MA, USA; Cat. No. A21202; working dilatation: 1:1000 and Alexa Fluor 546 nm donkey anti-guinea pig, ThermoFisher Scientific, Waltham, MA, USA, Cat. No. A11074, working dilution: 1:1000) for one hour at room temperature. After rinsing in PBS (3 × 10 min), the sections were covered with a polyethylene glycol/glycerine solution containing DABCO (Sigma, St. Louis, MO, USA). Negative controls, including pre-absorption for the neuropeptide antisera with appropriate antigens, as well as the omission and the replacement tests were performed to eliminate non-specific labelling.

Sections were then examined under an Olympus BX51 microscope and photographed with a digital monochromatic camera (Olympus XM 10) connected to a PC, equipped with the cellSens Dimension Image Processing software (Olympus, Hamburg, Germany). The number of PACAP-positive enteric neurons in all fragments of the studied GIT and the type of enteric plexus under investigation was established by counting at least 500 neurons with a clearly visible nucleus immunoreactive to Hu C/D (pan-neuronal marker) (the number of Hu C/D neurons was assumed as 100%). To avoid double counting of the same neuron, sections separated at least 200 μm away from each other were selected for the study. The obtained results were pooled, analysed statistically with Statistica 13 (Stat Soft Inc., Tulsa, OK, USA) and expressed as a mean ± standard error of mean (SEM). Significant differences were assessed with the Student’s t-test for independent samples (* *p* < 0.05, ** *p* < 0.01, and *** *p* < 0.001).

## 5. Conclusions

Streptozotocin-induced hyperglycaemia caused a significant increase in the population of PACAP-containing enteric neurons in the porcine stomach, small intestines and descending colon. The recorded changes may result from the direct toxic effect of hyperglycaemia on the ENS neurons, oxidative stress or inflammatory conditions accompanying hyperglycaemia and suggest that PACAP is involved in regulatory processes of the GIT function in the course of diabetes. PACAP has also been shown to have a beneficial effect in alleviating disorders of the diabetic retina and in the vascular complications of diabetes. Although natural PACAP is biologically unstable, pharmacological studies have led to the development of a metabolically stable PACAP38 analog, acetyl-[Ala15, Ala20]PACAP38-propylamide, which is a promising therapy for neurodegenerative diseases. Further research may establish its use in the alleviation of gastrointestinal dysfunction in the course of diabetes.

## Data Availability

Data is contained within the article.
